# Brain-inspired strategies for efficient artificial intelligence

**DOI:** 10.1016/j.mocell.2026.100365

**Published:** 2026-04-24

**Authors:** Min-Seo Kim, Hyoung F. Kim

**Affiliations:** 1 School of Biological Sciences, College of Natural Sciences, Seoul National University (SNU), Seoul 08826, Republic of Korea; 2 Interdisciplinary Program in Neuroscience, Seoul National University (SNU), Seoul 08826, Republic of Korea; 3 Institute for Data Innovation in Science, Seoul National University (SNU), Seoul, Republic of Korea

**Keywords:** Brain efficiency, Brain-inspired artificial intelligence, Dimensionality reduction, Funneling architecture, Value-based decision-making

## Abstract

Despite being constrained by a rigid skull and a fixed number of neurons, the brain excels in processing diverse information and making adaptive decisions with remarkable efficiency. This raises a central question at the intersection of neuroscience and artificial intelligence (AI): How does the brain achieve such high performance with limited physical resources? We addressed this question by examining the anatomical funneling architecture of the basal ganglia, a central hub for value-based decision-making. Through parallel processing, distinct circuits support cognitive flexibility and habitual stability, enabling efficient allocation of neural resources and context-sensitive engagement in specialized computations. In contrast, convergent processing compresses the input across circuits, allowing the efficient extraction of core information for decision-making, a form of quantitative efficiency that minimizes the number of neurons required. However, this compression can degrade the fidelity. To address this, the brain employs qualitative efficiency in which population-level neural patterns preserve fine-grained information and support generalization across similar contexts. Finally, we propose that the cortico-basal ganglia system achieves cognitive efficiency by funneling the anatomical structure and dimensionality reduction to optimize both performance and energy demands. These principles offer a biologically grounded framework for developing brain-inspired, resource-efficient artificial intelligence systems that balance generalization with precision.

## Graphical Abstract

**Figure:**
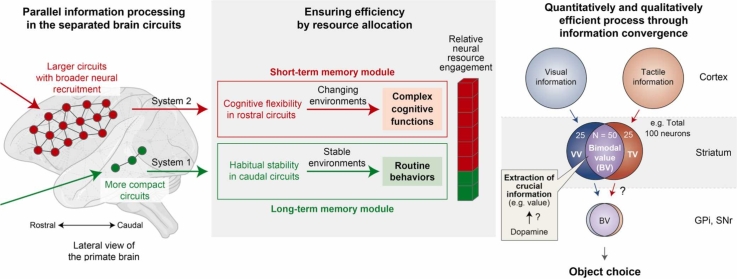

## INTRODUCTION

### The Brain: An Evolutionarily Advanced Information Processor and What We Can Learn From It

**Fig. 1:**
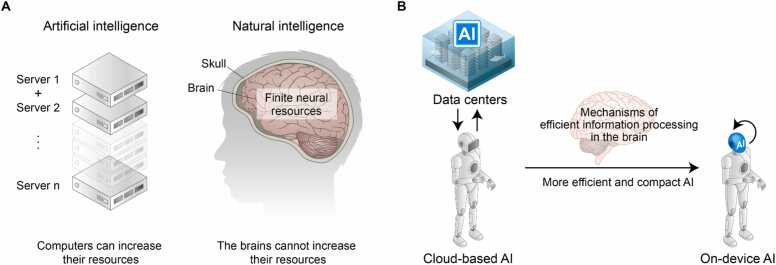
*Differences between artificial intelligence and natural intelligence.* (A) Resource expansion in artificial intelligence (AI) versus the limited resources of natural intelligence (NI). AI systems can scale up their computational power (left panel). However, the human brain is limited by biological anatomical constraints (right panel). (B) The implication of brain mechanisms for efficient information processing in AI. Understanding how the brain efficiently processes information under resource constraints, such as a limited number of neurons, provides insights that can guide the development of more efficient and compact on-device AI systems.

One straightforward approach for enhancing the computational power of AI is to increase the number of servers. In other words, AI systems can expand their resources to process information (Fig. 1A). However, unlike AI, which can scale its processing capacity by adding more hardware, the brain is confined to the skull, limiting its size and shape (Kaplan et al., 2020).

The primary unit of information processing in the brain is the neuron. A greater number of neurons per brain mass corresponds to a higher capacity for information processing, emphasizing the importance of the neuronal count (Herculano-Houzel, 2009). To increase the number of neurons in the brain, higher-level animals, such as primates, possess brain structures that differ significantly from those of lower-level animals like rodents (Zhang et al., 2023). For instance, in primates, an increase in the number of neurons leads to the formation of cortical sulci and temporal lobes, resulting in the characteristic convoluted shape of the primate cortex (Lee et al., 2023; Rakic, 1995). These structures are adapted to maximize the cortical surface area within the confines of the skull, which restricts outward expansion (Welker, 1990). Consequently, the brain is limited by the number of neurons that it can house, which imposes a cap on its information-processing resources.

Despite these constraints, the human brain excels in processing vast amounts of information, enabling optimal decision-making across various contexts (Bassett and Gazzaniga, 2011, Dosenbach et al., 2007). Our brain’s capability to achieve superior processing efficiency despite its limitations in terms of neuron count, resource expansion, and energy availability is remarkable, especially compared to current AI (Ren and Xia, 2024). The brain’s effectiveness in information processing suggests the existence of mechanisms that allow it to process external information efficiently and make the best decisions using limited resources. This capability likely evolved early in life as organisms rely on information processing for survival. However, the precise mechanism through which the brain achieves this efficiency remains largely unknown.

Currently, more advanced AI requires larger infrastructure, consumes more space, and demands more energy, which has led to the establishment of large data centers in unconventional locations, such as deep in the ocean or within cold mountain ranges (Allan, 2023, Downie, 2024, Ansett, 2024). Achieving greater efficiency in AI utilization is now critical not only for maximizing corporate benefits but also for conserving natural resources. Moreover, as robotics advances, AI systems need to be sufficiently compact to be integrated into robots that operate independently of network connections (Fig. 1B). While recent performance gains in AI systems largely stem from improved GPU architectures that perform parallel computations, simply scaling these physical engines is reaching its inherent limits. As AI continues to evolve, reducing its physical size and energy consumption becomes increasingly important. Hence, improving the limitations of existing physical engines requires a fundamental understanding of the brain’s neurophysiological information processing. We may find answers by studying the brain, which has evolutionarily developed efficient information-processing strategies (Zhang et al., 2020). Considering this, we explored how the structures of the cortico-basal ganglia system efficiently manage information processing using limited resources by drawing on recent findings.

### Efficient Resource Allocation in the Brain Through Parallel Circuits for Habitual Stability and Cognitive Flexibility

The dual-systems model popularized by Dr. Daniel Kahneman refers to 2 distinct systems in the brain (Kahneman, 2013, Kahneman, 2003). System 1 is characterized as automatic, fast, and often unconscious and is primarily responsible for generating habits and instinctual responses. In contrast, System 2 is deliberate, slow, and conscious and plays a crucial role in logical reasoning and adapting to new situations. In terms of efficiency, System 2 is generally considered to require more cognitive resources for continuous monitoring, flexibility, and overriding automatic responses when necessary, whereas System 1 enables automatic responses based on learned experiences, potentially making it highly efficient for routine tasks (Kahneman, 2013, Kahneman, 2003; Kim, 2021). In stable environments, System 1 is employed to execute optimal behavior with minimal energy consumption.

**Fig. 2:**
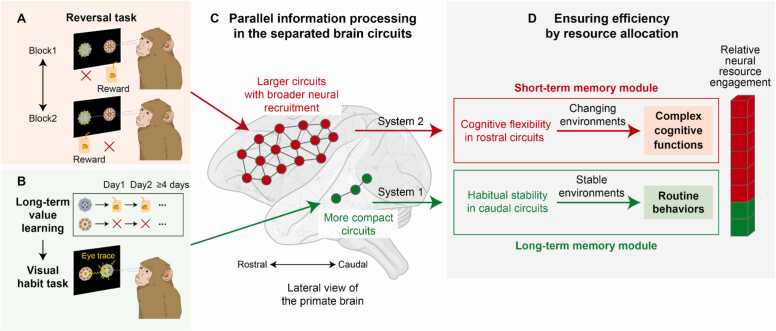
*Achieving efficiency by separating circuits for habitual stability and cognitive flexibility.* (A) The reversal task for examining cognitive flexibility mechanisms in the brain circuits. In this task, each fractal object is associated with either a reward or no reward within a block, and the object-reward association is reversed across blocks. Human or monkey subjects monitor object-reward associations and flexibly update the object's value when the reward changes, allowing them to select the correct answer and make faster saccades toward the rewarded object. (B) The long-term value learning in the visual habit task. Human or monkey subjects learn a fixed object-reward association for more than 4 days. After a retention period of at least 1 day following the final learning session, habitual gaze patterns are examined using the visual habit task. (C) Parallel information processing in the separated brain circuits. Cognitive flexibility, which is essential for adaptive behavior in the reversal task, is selectively processed in the rostral brain circuits. In contrast, habitual stability, which underlies habitual behavior in the visual habit task, is selectively processed in the caudal brain circuits. (D) Ensuring efficiency in stable environments while preserving resources for cognitive flexibility. Habitual stability is processed separately by simple circuits in the caudal brain regions, thus preserving sufficient resources for cognitive flexibility and higher-order mental functions.

The crucial question is whether corresponding biological neural circuits exist in the brain. Recent studies have found that these systems are divided within the structures of the basal ganglia along the rostral-caudal axis (Griggs et al., 2017; Hikosaka et al., 1999; Hikosaka et al., 2014; Jiang and Kim, 2018; Kang et al., 2021; Kim and Hikosaka, 2013; Kim and Hikosaka, 2015; Kim et al., 2014; Kim et al., 2017, 2015; Kim, 2021; Kim, 2025; Miyachi et al., 1997; Yamamoto et al., 2013). To examine neural responses under conditions requiring continuous monitoring and automatic responses, object reversal and visual habit tasks were used with macaque monkeys and human subjects, respectively (Fig. 2A and B).

In the object reversal task, as illustrated in Figure 2A, 2 objects were associated with different rewards, and the object-reward contingency was reversed across blocks, with each block consisting of 20-30 trials (Izquierdo et al., 2017; Kim and Hikosaka, 2013). For instance, in the first block, object A was paired with a liquid reward, whereas object B was not. This association was reversed in the next block without any explicit instruction. The monkeys were required to monitor reward associations, detect changes, and determine which object would provide reward in each block.

Notably, the rostral regions of the basal ganglia are selectively involved in processing these flexible changes of value (Kang et al., 2021; Kim and Hikosaka, 2015, 2013). Specifically, 6 structures within the basal ganglia system (the caudate, putamen, ventral striatum, globus pallidus externus, substantia nigra pars compacta, and substantia nigra pars reticulata) have been implicated in cognitive flexibility within their rostral regions (An et al., 2024; Hikosaka et al., 2014; Kang et al., 2021; Kim and Hikosaka, 2015, 2013; Kim et al., 2017, 2015; Yasuda and Hikosaka, 2015). Neurons in these structures encode the flexibly changing value in each block, showing higher responses to reversed high-valued objects than to low-valued ones, or vice versa. Notably, these flexible value-coding neurons are broadly distributed across the rostral regions of these structures, suggesting that more neurons are utilized to monitor and detect changes and encode optimal values in dynamic environments (short-term memory module) (Fig. 2C).

In the visual habit task, monkeys and humans were free to view fractal objects that had been previously associated with different rewards stably over more than 4 days using the long-term value learning task (Fig. 2B). Notably, no outcome was provided during the free-viewing trials, which facilitated automatic gazing at the objects based on stably sustained value memory (Kang et al., 2021; Kim and Hikosaka, 2013; Yamamoto et al., 2013; Yasuda et al., 2012). Interestingly, monkeys and humans frequently gazed more at objects that were previously associated with rewards, even in the absence of any reward outcomes, demonstrating an automatic gaze bias, known as a visual habit (Fernandez-Ruiz et al., 2001; Hwang et al., 2022; Kang et al., 2021; Kim and Hikosaka, 2015).

Electrophysiological studies in macaque monkeys have revealed that the long-term value memory of visual objects is selectively encoded in neurons located in the caudal regions of the basal ganglia, including the caudate, putamen, ventral striatum, globus pallidus externus, substantia nigra pars compacta, and substantia nigra pars reticulata (Kim, 2021, Kim and Hikosaka, 2015). Additionally, human fMRI studies have shown that the caudal regions of the ventral striatum and the caudate nucleus are involved in processing long-term memories of visual habits and motor skills (Kang et al., 2021; Miyachi et al., 1997; Seger, 2013; Choi et al., 2020; Farmani et al., 2024). Importantly, these neurons, which encode long-term value memory, are focally localized within the relatively smaller caudal regions of each basal ganglia structure, compared to the more widely distributed flexible value-coding neurons in the rostral regions (Fig. 2C). These functional and anatomical findings indicate that habitual stability is efficiently achieved with fewer neurons in the caudal regions of the basal ganglia structures (long-term memory module).

Overall, under stable conditions, the caudal circuits of the basal ganglia efficiently process habitual behavior based on long-term value memory using fewer neurons (System 1) (Fig. 2C and D). In contrast, under flexible conditions, the rostral circuits of the basal ganglia are engaged in processing adaptable behavior, relying on updating memory and utilizing a greater number of neurons (System 2) (Fig. 2C and D).

Together, these 2 systems enable animals to efficiently utilize limited neural resources depending on environmental demands. This functional distinction suggests that the brain may dynamically allocate neural resources between stable and flexible control systems (Fig. 2D). In stable environments, processing can be concentrated within relatively compact caudal circuits that support habitual responses, thereby minimizing metabolic and computational costs. In contrast, when behavioral flexibility and continuous monitoring are required, broader rostral circuits are recruited, reflecting a redistribution of neural resources to sustain adaptive computation.

### Quantitatively Efficient Process Using Information Extraction and Convergence

**Fig. 3:**
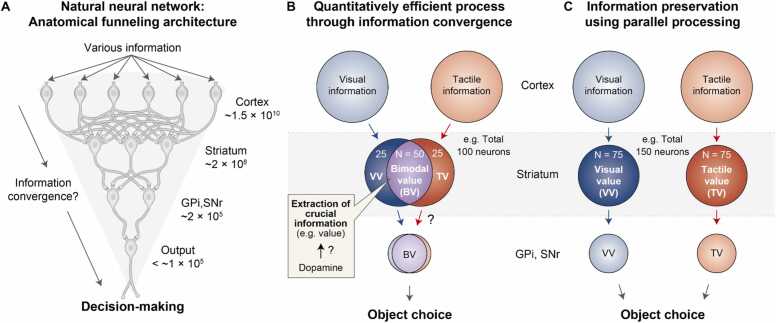
*Anatomical funneling architecture of the brain and its role in ensuring a quantitatively efficient process through information convergence*. (A) Anatomical funneling architecture in the natural neural network. The number of neurons progressively decreases through each neural layer, from the cortex to the striatum and then to the globus pallidus or substantia nigra; ultimately, processing occurs in the motor output regions for decision-making. GPi: Globus pallidus internus. SNr: Substantia nigra pars reticulata. (B) Quantitatively efficient process through information convergence. In an example of tactile and visual value processing, neurons in the striatum converge visual and tactile object information, extracting the most essential feature, namely, the object value required to select the most valuable object. This convergence process allows the brain to process information more efficiently, using fewer neurons compared to the parallel processing described in C. VV: Visual value. TV: Tactile value. BV: Bimodal value. (C) Parallel information processing.

A key constraint of the brain arises from the limited number of neurons within the skull, which challenges its ability to process vast amounts of information (von Bartheld et al., 2016). The number of neurons decreases as information flows from the cortex to successive structures in the basal ganglia (Bar-Gad et al., 2003; Morris et al., 2003; Nambu, 2011; Oorschot, 1996; Percheron et al., 1994; Yelnik, 2002). Figure 3A schematically illustrates this anatomical convergence and the concept of quantitative funneling across these regions. This leads to a critical question: How does the brain, with its layered cortico-basal ganglia circuits, manage to process diverse information and make decisions as the number of neurons progressively decreases?

One plausible explanation for this anatomical funneling structure is that information converges in single neurons (Bergman et al., 1998; Nambu, 2011). Previous studies by the Nambu group demonstrated anatomically overlapping motor regions within basal ganglia structures (Iwamuro et al., 2017; Nambu, 2011; Nambu et al., 2002). This supports the concept of a convergent process. However, this convergent process has the disadvantage of causing single neurons to lose specific details of information, such as which part of the limb is moving, while retaining only general information about the movement itself. A parallel-process hypothesis was proposed to address the issue of information loss (Alexander et al., 1986, Alexander et al., 1991, Alexander and Crutcher, 1990). In this parallel processing approach, each piece of information is processed intact across the circuits, ensuring that information is preserved at the single-neuron level. However, it requires more neurons than the convergent process. Therefore, it is crucial for the brain to optimize efficiency by utilizing fewer neurons while retaining the critical information necessary for movement and decision-making. This necessity implies the existence of mechanisms within the brain that allow for the extraction of crucial information and efficient processing with fewer neurons.

This extraction, through a convergent process, has been observed in the primate striatum of the basal ganglia when monkeys choose the most valuable objects (Hwang et al., 2024). To examine this value convergence process, monkeys were trained to associate different rewards with visual and tactile objects, and their neuronal responses to these objects were recorded as they identified the most rewarding object. Two possible outcomes emerge based on the convergent and parallel process hypotheses. If the tactile and visual values converge, a single neuron encodes both values, facilitating efficient object choice with fewer neurons (Fig. 3B). Conversely, if the values are not abstracted, a single neuron encodes either tactile or visual values, thereby preserving both modality and value information (Fig. 3C).

Single neurons in the primate striatum encode a converged form of values from diverse sensory inputs (Fig. 3B). Notably, more than half of the striatal neurons were identified as bimodal value-coding neurons (Hwang et al., 2024). This convergence process enhances the efficiency of the value coding by utilizing a limited number of neurons. As the example in Figure 3B depicts, if there are 50 bimodal value-coding neurons among 100 value-coding neurons, then 75 neurons are involved in processing visual values and 75 neurons in processing tactile values. In the parallel process, however, 75 modality-selective value-coding neurons are required for each value process, resulting in a total of 150 neurons, 50 more than in the convergent process (Fig. 3C). To make a choice based on the object value, such as grasping it or not, the brain may only need to use value information without modality details. This allows 50 additional neurons to remain available for other tasks.

As described in the example of making optimal decisions based on object value, the basal ganglia system extracts crucial data, specifically value information, from visual and tactile objects while disregarding their modality. Dopamine (Fig. 3B) may play a critical role in extracting value from both modality and value information because dopamine neurons send reward prediction error signals and are involved in synaptic plasticity (Calabresi et al., 2007; Jay, 2003; Joshua et al., 2009; Kim et al., 2015; Reynolds and Wickens, 2002; Schultz, 1998; Yagishita et al., 2014). This convergent process minimizes the use of limited neural resources and facilitates efficient value-guided decision-making.

### Qualitatively Efficient Information Process Using Population Neural Patterns: The Dual Information Encoding Strategy

In the brain's extraction of information using fewer neurons, we might ask whether a quantitatively efficient process through convergence inevitably compromises the information quality. Does the brain lose some information to achieve quantitative efficiency, or is all the information preserved in a different form within the brain?

**Fig. 4:**
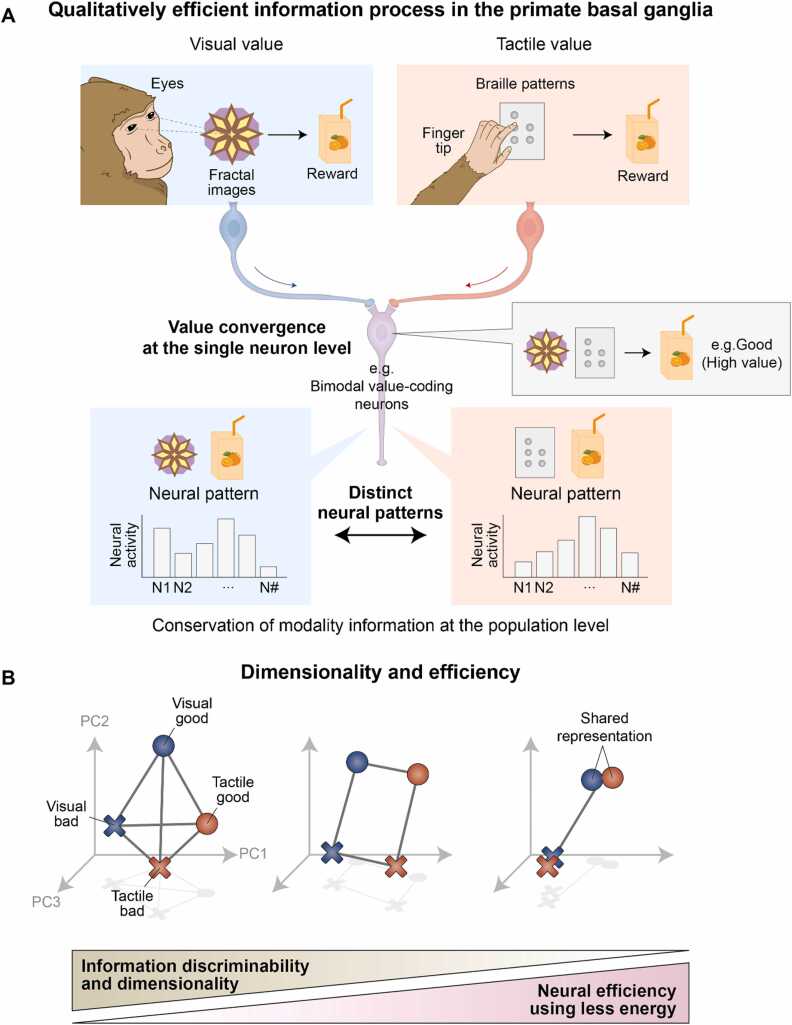
*Qualitatively efficient information process through neural population coding and dimensionality reduction*. (A) Qualitatively efficient information process in the primate basal ganglia. Modality information discarded at the single-neuron level through value extraction and convergence is preserved at the neural population level, demonstrating qualitatively efficient information processing with fewer resources. N# indicates each individual neuron. (B) Dimensionality and efficiency. Greater shared representation reduces dimensionality and resource demands for information processing. N1, N2, and N3 represent the imaginary axes corresponding to the firing rates of individual neurons.

Recent studies analyzing neural ensembles have addressed this question under conditions of visual and tactile value processing in macaque monkeys (Hwang et al., 2024). As described in Figure 3B, bimodal value-coding neurons encoded value information regardless of which modality was associated at the single neuron level. However, at the population neuron level, these bimodal value-coding neurons represent both modality and value information (Fig. 4A) (Hwang et al., 2025). This example demonstrates that the brain extracts crucial information through convergence at the single-neuron level while preserving all relevant information at the population neuron level to maintain its quality (qualitatively efficient information processing). This dual information-encoding strategy highlights how the brain, including the basal ganglia, balances efficiency by maintaining detailed information across different levels of neural processing.

### Reducing the Dimensionality of Neural Geometry Through Shared Representations for Efficient Information Process

Representing information through distinct neural population patterns can preserve the information quality, and a larger repertoire of such patterns allows for the retention of finer details. For example, 4 variables (tactile-good, tactile-bad, visual-good, and visual-bad) can be represented separately in the principal component (PC) space through 4 distinct neural patterns in the brain (Fig. 4B, left diagram). However, if these representations are grouped by value (good and bad), the brain will generate only 2 neural patterns, one shared representation for each value, regardless of the modality (Fig. 4B, right diagram). Therefore, when neural geometry includes more shared representations, its dimensions decrease (Fig. 4B), suggesting that both dimensionality and information discriminability may decrease (Ostojic and Fusi, 2024).

In the context of Shannon’s information theory, a greater number of different neural patterns corresponds to a higher entropy level, which typically requires higher energy consumption (Amalric and Cantlon, 2023; Ostojic and Fusi, 2024; Rajaram and Castellani, 2016; Shannon, 1948). Considering the energy consumption at the neuron level, generating a greater number of different neural patterns may require more energy because transitioning from one brain state to another necessitates synaptic changes and maintenance, such as phosphorylation-driven alterations in ion channel responses and translation-dependent structural changes in synapses, all of which demand ATP supply (Buffington et al., 2014; Costa-Mattioli et al., 2009; Harris et al., 2012; Ismailov and Benos, 1995; Kandel, 2001; Lee, 2006; Schacher et al., 1988; Sweatt and Kandel, 1989). This differs from the condition in which the brain reduces the number of neurons involved in information processing through redundancy reduction, as previously suggested (Lennie, 2003). As illustrated in Figure 4B (middle panel), neural representation patterns with an appropriate number of neurons achieved good discrimination of information details.

Sharing neural representations could save energy compared with maintaining completely distinct neural representation patterns, as less energy is required to adjust synapses for different situations when representations are shared (Bar-Gad et al., 2003, Harris et al., 2012). However, while using shared representations can conserve energy during information processing, it often compromises discriminability (Fig. 4B, right diagram). Therefore, the brain must find a balance between efficient encoding, which conserves energy, and preserving information discriminability. However, the mechanisms underlying the energy-saving process remain unclear. Empirical evidence is crucial for understanding how the brain navigates this trade-off and for providing insights into the fundamental principles of neural efficiency.

### Dimensionality Dynamics in the Cortico-Basal Ganglia System: Models of Neural Representation

**Fig. 5:**
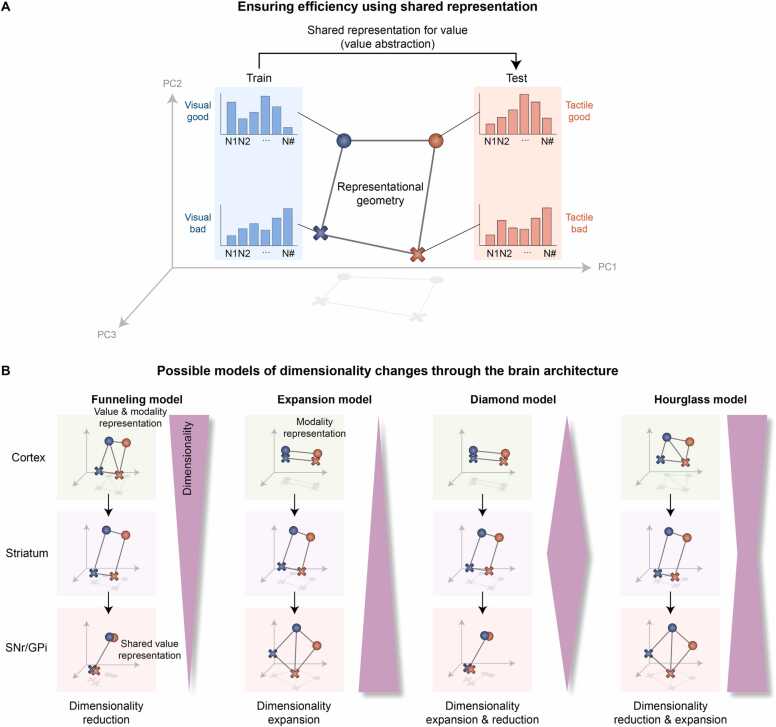
*Plausible dimensionality reduction through the brain architecture.* (A) Ensuring efficiency through shared representation. Principal component analysis (PCA) visualizes the representational geometry of neurons. PC: Principal component. (B) Four models of dimensionality transformations through anatomical funneling architecture. In the Funneling model, information dimensionality progressively decreases across successive neural layers, from the cortex to the striatum and then to the SNr or GPi. This enables compressed representations to guide cognitive behavior. In contrast, the Expansion model features a stepwise increase in dimensionality, with additional information such as modality or salience more incorporated at each stage. The Diamond model shows an initial expansion followed by a reduction in dimensionality downstream. Conversely, the Hourglass model begins with high-dimensional cortical input, compresses information at the level of the striatum, and re-expands it in downstream structures such as the SNr and GPi.

Notably, recent studies by the Fusi group have demonstrated this shared representational geometry in higher-order brain regions, including the cortical and limbic structures (Bernardi et al., 2020; Courellis et al., 2024; Rigotti et al., 2013). Furthermore, this shared representation was also observed in the primate basal ganglia (Hwang et al., 2025). As illustrated in Figure 5A, the 4 variables can be separately decoded with distinct neural population patterns, but the patterns for good and bad values are sufficiently similar to be shared across both the tactile and visual modalities. Specifically, this shared geometry demonstrates that neural populations do not merely contain diverse information but organize it into a structured, low-dimensional manifold. The preservation of this representational geometry allows the brain to employ an internal, abstracted representation of value that remains invariant to whether it is perceived through visual or tactile senses. This suggests a possible strategy that brain structures, from the cortical to deep brain regions, employ dimensional reduction through partially shared representations to achieve energy-efficient information processing (Fig. 5B, Funneling model). This partially shared representation allows the brain to achieve efficient information encoding by reducing the dimensionality of the neural geometry while preserving information detail.

However, we can also consider alternative models in which neural populations process information differently from the Funneling model described above. These models provide distinct frameworks for understanding how the dimensionality of neural representations is transformed across successive stages of the basal ganglia. For example, in the context of value and modality information, the Expansion model proposes that value signals are first introduced at the level of the striatum, while more detailed information, such as modality and salience, is subsequently encoded at downstream structures, including the SNr and GPi (Fig. 5B, Expansion model). In this model, the dimensionality of information increases progressively through each stage of the cortico-basal ganglia circuit. The Diamond model also posits that value information is initially represented in the striatum. However, in this case, downstream structures such as the SNr and GPi do not further process additional features like modality (Fig. 5B, Diamond model). As a result, the dimensionality of information first expands and then contracts along the circuit. Lastly, the Hourglass model suggests that both value and modality information are initially processed in cortical areas. These signals are then funneled into a shared, lower-dimensional representation in the striatum, similar to the Funneling model. However, at the level of the SNr and GPi, additional information is reintroduced, leading to an expansion in dimensionality beyond that seen in the striatum (Fig. 5B, Hourglass model). Thus, in the Hourglass model, dimensionality is first reduced and then expanded, reversing the pattern proposed in the Diamond model.

To elaborate on the functional implications of these representational transformations, each model proposes distinct computational principles that may operate under biological constraints (Table 1). Importantly, these models are not presented as established mechanisms but as conceptual frameworks for interpreting how dimensionality may be reorganized across cortico-basal ganglia circuits.

**Table 1:** Conceptual models of dimensionality transformation in neural circuits and their implications for AI.

Model	Dimensionality change	External input required	Information representation	AI analogy
Funneling	Progressive reduction	Not necessarily	Information is progressively compressed from cortex → striatum → SNr/GPi into a shared representation.	On-device inference modules
Expansion	Progressive increase	Yes	Modality (cortex) expands with value (via SNc) in striatum, and integrates context (via hyperdirect pathway) at SNr/GPi.	Kernel methods; Random feature expansion
Diamond	Increase → reduction	Yes	Striatum initially expands dimensionality to evaluate features, but SNr/GPi compresses it to finalize action choice.	Deep feedforward networks
Hourglass	Reduction → re-expansion	Yes	Funneled into a lower-dimensional representation in striatum, then expanded at SNr/GPi to execute specific plans.	Encoder–decoder architectures

First, the Funneling model proposes that neural circuits may progressively reduce representational dimensionality through convergent projections (Fig. 5B). Anatomical convergence from the cortex to the striatum and further to the SNr/GPi provides a structural substrate for such compression. Rather than implying a strict quantitative relationship between neuron number and hierarchy, this model suggests that shared representations can abstract value information across sensory modalities, enabling generalized value-based decision-making with reduced redundancy.

Second, the Expansion model considers the possibility that representational dimensionality may increase at certain processing stages (Fig. 5B). While cortical inputs may initially encode modality-specific signals, additional value-related dimensions could be incorporated within the striatum through dopaminergic reward prediction error signals from the SNc. Furthermore, downstream nuclei such as the SNr and GPi may integrate additional task-relevant variables, including salience or contextual inputs, through interactions with circuits such as the hyperdirect cortico-subthalamic pathway. In this view, dimensional expansion reflects the incorporation of additional behaviorally relevant inputs, such as value, salience, or contextual signals, thereby enriching the representational space available for action selection. Such expansion may enhance the separability of competing action representations and improve the accuracy and robustness of final action selection under complex or ambiguous conditions.

Third, the Diamond model posits a transient expansion of dimensionality followed by downstream compression (Fig. 5B). Here, intermediate structures may temporarily increase representational richness to evaluate multiple features of value and modality. Such intermediate expansion may enhance the separability of competing action representations, thereby improving classification and decision accuracy under complex conditions. Subsequent compression at output nuclei such as the SNr and GPi may then retain only the decision-relevant components required for action selection, reducing redundancy and conserving neural resources. This transition from high- to low-dimensional representations enables flexible feature evaluation while restoring efficiency at the motor output stage.

Finally, the Hourglass model describes an initial reduction of dimensionality followed by re-expansion (Fig. 5B). Diverse cortical inputs containing value and modality information may first converge into a compact, low-dimensional representation within the striatum, preserving only the core task-relevant features. This compressed latent state can then be re-expanded in downstream nuclei to support more detailed discrimination and context-specific action selection. In this framework, dimensional re-expansion enables refined classification and flexible motor implementation while maintaining efficiency at the intermediate processing stage.

Taken together, these 4 models illustrate that cortico-basal ganglia circuits may employ multiple strategies of dimensionality reduction and expansion, rather than relying exclusively on monotonic convergence. Such flexibility in representational organization may provide a broader computational repertoire for balancing stability, adaptability, and energy efficiency.

## CONCLUSION AND FUTURE RESEARCH

Previous studies have highlighted the ability of the brain to efficiently process information using 3 complementary mechanisms: parallel processing, convergent processing, and population encoding. Collectively, these mechanisms enable the brain to manage complex information with limited resources, balancing efficiency and precision.

The basal ganglia utilize 2 distinct circuits along the rostral-caudal axis to balance cognitive flexibility and habitual stability (Fig. 2). This dual-circuit organization enables animals to optimize energy use by selectively engaging the appropriate circuit along the rostral-caudal axis of the brain in response to contextual demands (Kim, 2025). This mechanism highlights the central role of the basal ganglia in enabling adaptive and goal-directed behavior by allocating neural resources to support cognitive flexibility. Although the rostral–caudal axis provides a foundational framework for functional organization, our review extends this framework by linking it to specific efficiency strategies that may sustain the relatively high resource demands of rostral circuits involved in cognitive flexibility. Understanding how quantitative and qualitative efficiency are achieved within the rostral system under intrinsic physical constraints provides important insights into the neural principles underlying efficient information processing during behavioral adaptation.

The convergent process observed in the basal ganglia quantitatively supports efficient information processing (Hwang et al., 2024). By extracting crucial information such as values from different modalities through the convergent network, the system minimizes the total number of neurons required for decision-making, thereby conserving energy (Fig. 3). A critical question remains: what mechanisms does the brain use to determine which information is necessary and which is not? One plausible hypothesis is that heterosynaptic manipulation, primarily mediated by dopaminergic neurons from the SNc and ventral tegmental area (VTA), may regulate the weighting of information based on context (Fig. 3, Fig. 5) (Ambrosi and Lerner, 2022; Eyny and Horvitz, 2003; Haber et al., 2000; Joel and Weiner, 2000; Nakahara et al., 2004; Nakamura and Hikosaka, 2006; Shen et al., 2008).

Extracting essential information often results in the loss of other information that may be relevant to decision-making. To address this limitation, neurons in the striatum retain non-essential information for decision-making within neural population response patterns while preserving information quality (Fig. 4A). Furthermore, these neural patterns are not randomly distributed in the PC space; rather, the neural representation is shared, which reduces representational dimensionality (Fig. 4B). This dimensionality reduction is a potential strategy for optimizing the limited resources in the brain. Investigating changes in the dimensionality of information representation across natural neural layers in the brain, including the cortex, striatum, and output structures such as the SNr and GPi, is crucial for understanding processing within the basal ganglia (Fig. 5B).

Importantly, beyond advancing biological understanding, the 4 models of dimensionality dynamics in the basal ganglia may offer conceptual guidance for the development of brain-inspired AI architectures.

First, the Funneling model illustrates how progressive dimensionality reduction can support efficient computation by minimizing redundancy while preserving decision-relevant information. Such an architecture may be particularly suitable for on-device AI systems, where computational resources and energy budgets are strictly limited and efficient inference is essential.

Second, the Expansion model demonstrates how low-dimensional value signals can be transformed into richer representational spaces through the integration of additional task-relevant inputs. In artificial systems, this parallels kernel methods and random feature expansion, which project data into higher-dimensional spaces to enhance separability (Boser et al., 1992; Rahimi and Recht, 2007).

Third, the Diamond model describes a transient expansion of representational dimensionality followed by compression into a decision-relevant output. This structure resembles deep feedforward networks, where intermediate layers initially expand the dimensionality to evaluate complex features, and subsequent layers compress the information to finalize the output (LeCun et al., 2015; Goodfellow et al., 2016).

Finally, the Hourglass model parallels encoder–decoder architectures widely used in deep learning (Cho et al., 2014). By compressing inputs into a shared low-dimensional latent representation to extract core features and subsequently expanding them to generate task-specific outputs, this structure illustrates how complex outputs can be produced efficiently without maintaining high dimensionality throughout the entire processing pipeline.

Among the proposed models, the Funneling model appears broadly consistent with the anatomical organization of cortico-basal ganglia circuits. Such organization may support a gradual reduction in representational dimensionality while maintaining decision-relevant information at the population level, offering a plausible interpretation of how neural systems balance information integration with metabolic and computational efficiency under biological constraints. However, it remains possible that additional inputs, such as those conveyed through the hyperdirect cortico-subthalamic pathway, introduce new task-relevant information that dynamically reshapes representational dimensionality at certain processing stages. Further empirical investigation will be required to determine how dimensionality is regulated across these interacting pathways under varying behavioral demands.

Additionally, future studies are expected to raise other fundamental questions. Is there any functional advantage in encoding information in higher-dimensional spaces? What are the causal effects of impairments in these neural population patterns? What mechanisms regulate the balance between processing efficiency and information accuracy? Addressing these questions will not only deepen our understanding of neural information processing in the brain but also reveal the efficient mechanisms underpinning natural intelligence. These insights provide a foundation for developing efficient brain-inspired AI.

## CRediT authorship contribution statement

**Min-Seo Kim:** Writing – review & editing. **Hyoung F. Kim:** Writing – review & editing, Writing – original draft, Conceptualization.

## Declaration of Competing Interest

The authors declare that they have no known competing financial interests or personal relationships that could have appeared to influence the work reported in this paper.
